# Psych Surf Soc: A Preliminary Study Evaluating the Effect of Surfing on the Wellbeing of Resident Doctors

**DOI:** 10.1192/bjo.2026.11298

**Published:** 2026-06-30

**Authors:** Dominic Menham

**Affiliations:** Avon and Wiltshire Mental Health Partnership, Bristol, United Kingdom

## Abstract

**Aims:**

The positive effects of exercise on wellbeing are well established, and an increasing body of evidence suggests feelings of nature connectedness and exposure to ‘blue space’ (aquatic environments) may also benefit wellbeing. Maintaining wellbeing is important for resident doctors who face high levels of academic and clinical stress, risking burnout. The author hypothesises that surfing, which combines exercise, nature immersion, and blue space, may benefit wellbeing. This preliminary research aims to evaluate resident doctor perceptions of their wellbeing, and how they felt it was impacted by surfing.

**Methods:**

Participants were recruited from the ‘Bristol Psych Surf Soc’, a surfing society for doctors. Respondents were predominantly psychiatry trainee doctors, although also includedwere foundation doctors and trainee doctors in other specialties. A six-question digital questionnaire measured how the doctors rated their wellbeing, how recently they had last surfed in the ocean, their perceptions of the effect of surfing on their wellbeing, beliefs on how surfing affected work-related stress, how surfing impacted feelings of connection to nature, and possible mechanisms by which surfing affected wellbeing. Responses were a mix of numerical rating scales, 5-point Likert scales, and free-text boxes.

**Results:**

15 responses were gathered. The mean wellbeing score was 7/10 (1=low, 10=high), with a range from 3–9. There was no correlation between time from last surf and wellbeing score (R²=0.13). 93% of respondents rated surfing as having a positive or strongly positive effect on their wellbeing, and 80% felt that surfing specifically reduced work-related stress. 93% of respondents reported surfing increased feelings of connection to nature. Free-text themes included mindfulness during surfing and increased feelings of connection to nature being beneficial for wellbeing, and surfing acting as a ‘reset’ with a subsequent uplift in feelings of wellbeing.

**Conclusion:**

While there was no clear correlation between recency of surfing and ratings of wellbeing, this preliminary research shows that participants nearly unanimously reported that surfing has a positive impact on their wellbeing and reduces work-related stress. Potential mechanisms for this effect are suggested by the free text responses. Limitations include the small sample size, potential sample bias due to participants being selected from a society for people who are enthusiastic about surfing, and a lack of a control group who did not surf. Further research could assess in more depth which aspects of surfing might provide the perceived positive impact on wellbeing, and whether surfing in wave pools could have similarly positive impacts.

